# Twists and Turns: A Chiropractic Roller Coaster Experience Unraveling the Intricacies of Bilateral Vertebral Artery Dissection

**DOI:** 10.7759/cureus.54990

**Published:** 2024-02-26

**Authors:** Satori Iwamoto, Megan D Hsu, An Phuc D Ta, Alexis Leo, Harrison Chu, Gary Chu

**Affiliations:** 1 College of Medicine, California Northstate University, Elk Grove, USA; 2 Internal Medicine, California Northstate University, Elk Grove, USA

**Keywords:** roller coasters, headaches, dual anti-platelet therapy, chiropractic manipulation, vertebral artery dissection

## Abstract

The primary insult in vertebral artery dissections (VADs) involves a tear in the vertebral artery intima, resulting in potential thrombus formation and an elevated risk of cerebrovascular events, such as stroke. Despite its relatively low overall incidence rate, VADs contribute to a significant proportion of ischemic strokes within the younger population. VAD has been associated with various risk factors including but not limited to neck trauma from chiropractic manipulation and significant neck movements. Our patient initially presented with a worsening occipital headache but was discharged due to the absence of any red-flag symptoms. However, the patient shortly returned to the ED upon worsening symptoms, and despite the lack of apparent neurological deficits, the patient's history of cervical spine manipulation and exposure to neck trauma risk activities (roller coaster riding) increased suspicion for VAD. This case highlights the importance of considering VAD as a differential diagnosis in young patients presenting with unexplained headaches and neck pain following events that exert stress on the vertebral arteries, such as roller coaster rides and chiropractic neck manipulation. When managed properly, the long-term prognosis of VAD is generally favorable; however, the risk of recurrent dissection and stroke still remains. Thus, this case further emphasizes the need for timely intervention and the role of dual anti-platelet therapy (DAPT) in the management of VAD to prevent further complications such as stroke.

## Introduction

Vertebral artery dissections (VADs) involve tears in the vertebral artery media, allowing blood to flow into the arterial wall. This results in the formation of intramural thrombus which can occlude blood vessels and present an increased risk of cerebrovascular accidents (CVA), commonly known as strokes [1]. VADs account for approximately 2% of all ischemic strokes, with a higher prevalence ranging from 10% to 25% in middle-aged and younger patients (30-45 years of age). The combined incidence of both VADs and carotid artery dissections is estimated to be 2.6 per 100,000, with carotid artery dissections 3-5 times more common. Chiropractors often dismiss the association between spinal manipulation and VAD by attributing the dissection as the cause of neck pain and the primary reason patients seek out chiropractic care. Thus far, there is no conclusive evidence establishing a direct link between chiropractic manipulation and VAD, only a causative association [2]. 

Various risk factors associated with VAD include yoga, nose blowing, judo, wrestling, hypertension, being of the female gender or Asian descent, and vasculitis [2]. Neck distortion, including bending of the neck during chiropractic manipulation, can lead to dissection and potentially complicate matters with a stroke. Neck trauma from incidents like whiplash in vehicle accidents or rapid head movements also pose a risk. Interestingly, spinal manipulative therapy (SMT) was found to be an independent risk factor for VAD. SMT is a technique where practitioners apply pressure to different joints in the spine [3]. This means that people who undergo SMT are at risk of experiencing VAD. Additionally, connective tissue disorders such as vascular Ehlers-Danlos syndrome and fibromuscular dysplasia can also increase the risk of VAD. One study found significant findings (p<0.001), indicating a higher prevalence of connective tissue abnormalities in patients experiencing spontaneous cervical artery dissections (sCeAD) compared to those with non-CeAD. The mean number of pathologic findings was 4.5±3.5 in the sCeAD group compared to 1.9±2.3 in the non-CeAD group [4].

Patients with VAD commonly exhibit acute symptoms, such as unilateral pain in the occipital-cervical area of the neck or head accompanied by headache, often following some form of neck trauma before the onset of pain. Although neurological deficits may present later, the most prevalent symptoms include dizziness, vertigo, diplopia, dysarthria, unilateral hearing loss, or ataxia [2]. Generally, headaches can be characterized by severe pain located around the eye or one side of the face, sometimes accompanied by other symptoms such as rhinorrhea or ptosis [5]. While headaches can affect both adults and children, adult women tend to be affected almost three times as much as men [5]. Various triggers such as emotional or environmental stress, sensory overload (visual and auditory), muscle-straining postures, or secondary disorders can cause headaches. Further classifying headaches into primary types (which include migraines, tension-type, or cluster) or secondary types can help determine appropriate work-up and management strategies [6]. 

When dealing with headaches caused by secondary disorders, it is important to recognize "red-flag" symptoms that include thunderclap headaches, focal neurological symptoms, increased frequency or severity of headaches, papilledema, and headaches arising from head/neck trauma or an underlying medical condition [6]. In cases where patients present with a nonspecific headache following neck trauma, VAD has been traditionally diagnosed with a CT angiogram due to its high sensitivity. However, MR angiograms have now become the gold standard test in terms of efficacy and superior imaging [7,8]. 

Patients diagnosed with VAD may experience complications including brain ischemia, ischemic strokes, and transient ischemic attacks. Some individuals exhibit signs of subarachnoid hemorrhage (SAH) along with symptoms like head and neck pain, pulsatile tinnitus, and cervical radiculopathy. VAD is a potentially disabling and often underdiagnosed cause of stroke that predominantly affects young adults. Furthermore, VAD was observed more frequently in the V2 or V3 vertebral artery segments (C2-C5 vertebrae) than the V1 or V4 segments, with the majority of patients presenting with posterior circulation ischemia and associated symptoms such as stroke, neck pain, or occipital headache [2]. 

Survivors of the initial dissection have a good prognosis with 80% having full recovery; however, around 10% of patients do not survive the initial dissection [2]. The primary goal of treatment is to prevent complications, in particular strokes, through anticoagulation with heparin. Thrombolytic therapy can be started within 4.5 hours from symptom onset if there are no contraindications. The use of endovascular therapies remains controversial but is considered a treatment option for patients [9]. Dissections tend to heal on their own, but blood-thinning medications such as dual anti-platelet therapy (DAPT) (aspirin and clopidogrel) may be prescribed to reduce the risk of blood clots [10]. In cases where standard procedures are not effective, interventions such as endovascular embolization, angioplasty, and intracranial stenting may be performed [11].

In this case report, we describe a young healthy female who presents with a headache due to bilateral VAD following a combination of two trauma-inducing events: chiropractic manipulation of her neck and subsequent multiple roller coaster rides. This case highlights the importance of VAD in the differential diagnosis of headache and neck pain in addition to the potentially elevated risk of VAD associated with procedures or events that elicit neck trauma.

## Case presentation

A 30-year-old Caucasian female with no significant past medical history was brought in late at night by a family member to the ED for a persistent headache that had been worsening over the past week. She described the pain as a burning sensation, rating it 6 out of 10 for headache severity, located in the right occipital region. The initial onset was sharp and sudden, originating in the neck and radiating up to the top of her head. Although the pain fluctuated in intensity, reaching peaks and subsiding, it never fully resolved. The patient denied any family history of SAH, intracranial aneurysm, or connective tissue disorders. She did not have a fever, nausea, vomiting, light/sound sensitivity, cough, congestion, rhinorrhea, sore throat, recent travel, or weakness. Additionally, there were no "red-flag" symptoms, such as thunderclap pain, neck stiffness, systemic or neurological symptoms suggestive of SAH, meningitis, or mass, respectively. Upon examination, vitals showed a blood pressure of 156/93 mmHg (subsequent blood pressure readings were normal without intervention), pulse of 81 bpm, temperature of 98°F, respiratory rate of 20/min, and oxygen saturation of 98% on room air, with a BMI 24.98 kg/m^2^. The head, eyes, ears, nose, and throat exam was normal and was normocephalic atraumatic, extraocular movements were intact, pupils were equally round and reactive to light, there was no temporal tenderness, tympanic membranes were clear, there was no sinus tenderness, and mucous membranes were moist. The neck exam showed a supple neck with a full range of motion and no thyromegaly. Neurological exam showed intact cranial nerves 2-12, non-focal findings, intact sensation to light touch, 5/5 strength in all four extremities, a steady gait, no pronator drift, and intact coordination. Empirical treatment was initiated with intravenous analgesics (diphenhydramine, metoclopramide, and ketorolac), and the patient was discharged that night with instructions to return to the ED if the pain worsened. 

**Figure 1:**
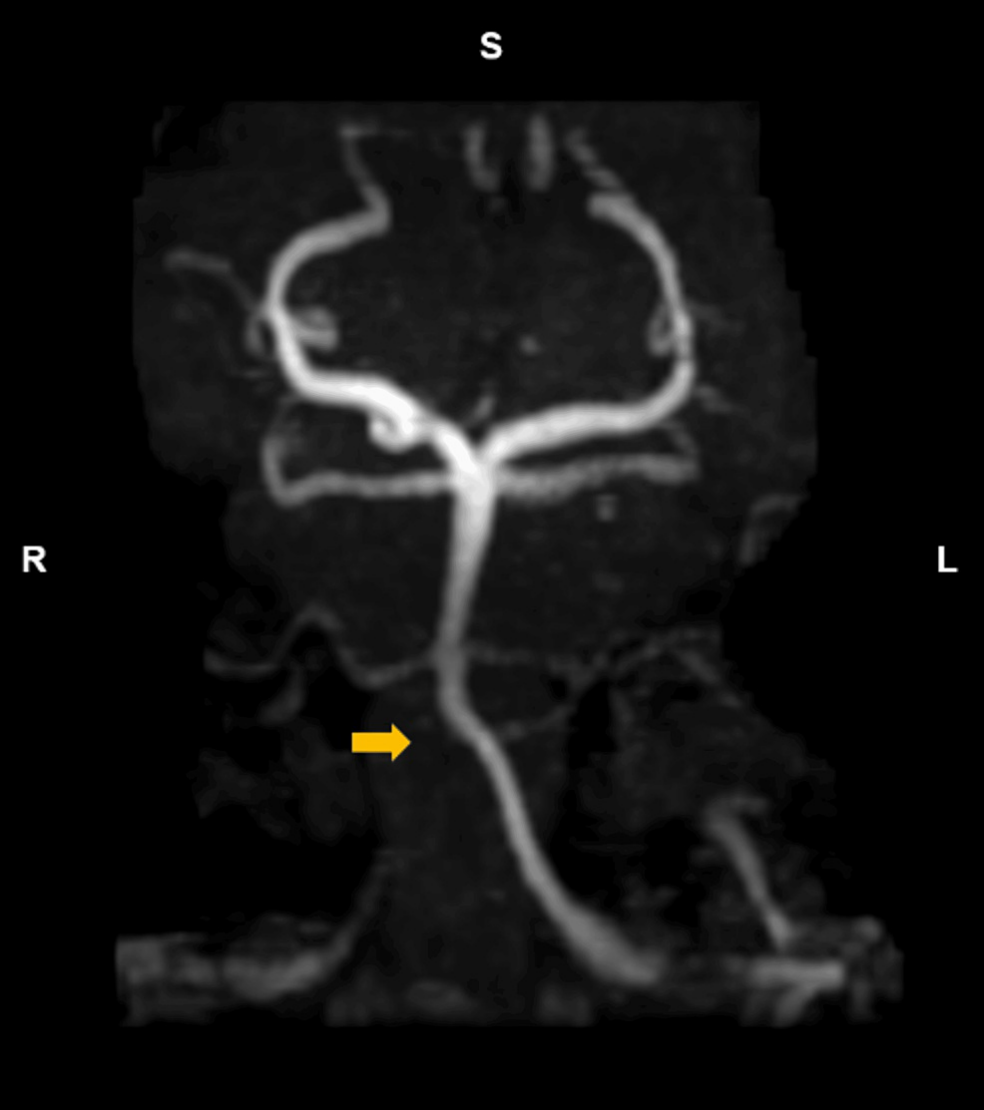
MR angiography shows irregular right vertebral artery flow void (indicated by gold arrow) as it crosses the dural margin.

**Figure 2:**
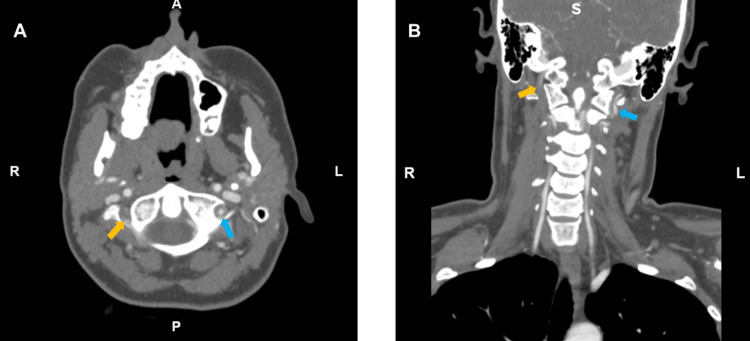
(A) Transverse plane and (B) coronal plane CT angiogram of the neck show severe stenosis of both superior cervical vertebral artery segments starting at the C2 vertebrae level, with thin residual hairline lumens seen at the C1 vertebrae (indicated by light blue and gold arrows). The left vertebral artery dissection ends just prior to the dural margin (indicated by light blue arrow), and the right vertebral artery dissection crosses the dural margin and involves the proximal intradural right vertebral artery (indicated by gold arrow).

The patient returned to the ED early the next morning, just six hours after her initial discharge, reporting worsened pain now located in the front of her head, radiating down her neck. The severity of her pain had also increased to a 7 out of 10, characterized by a throbbing and stabbing sensation. While she experienced nausea, she denied any vomiting, unilateral weakness, dizziness, recent head injuries, fever, sweats, or chills. Upon further investigation, it was discovered that the patient had seen a chiropractor about a month prior for a neck manipulation procedure for her chronic neck pain, and a week or two later, she had ridden multiple roller coasters at a local amusement park. She did not recall any other inciting events, and her physical activity consisted of walking, without participation in any contact/extreme sports or weightlifting. There were no notable changes to vitals and physical exam findings from her previous visit. Laboratory tests, including complete blood count (CBC), complete metabolic panel (CMP), human chorionic gonadotropin (HCG), and erythrocyte sedimentary rate (ESR) were all within normal range. Imaging findings with non-contrast CT and MRI were negative for stroke or other acute abnormalities (normal diffusion-weighted imaging findings), but MR angiography showed irregularities in the right vertebral artery flow (Figure 1). Per radiology, "flow void appears irregular as it crosses the dural margin in the V3 and V4 segments, and the possibility of intrinsically T1 hyperintense intramural thrombus on sagittal T1-weighted images, raising concerns for a dissection. The left vertebral artery dissection appears to end just before the dural margin. The right vertebral artery dissection appears to cross the dural margin and involve the very proximal intradural right vertebral artery, as well." Subsequent CT angiogram of the neck with contrast revealed significant bilateral VAD extending from the superior cervical segments and to the dural margin (Figure 2). Radiology noted "severe/critical stenosis of both superior cervical vertebral artery segments" with "both vertebral artery dissections begin[ing] at the C2 level. There was mild-to-moderate narrowing of the left vertebral artery at the C2 and C1-2 levels and severe narrowing of the right vertebral artery at the C2 and C1-2 levels. Only thin residual hairline lumens were seen at the C1 level, just before the dural margin. The left vertebral artery dissection appears to end just before the dural margin. The right vertebral artery dissection appears to cross the dural margin and involve the very proximal intradural right vertebral artery, as well." Neurology and neurointerventional radiology were consulted, leading to the initiation of the recommended DAPT (clopidogrel 75 mg and aspirin 81 mg) daily for three weeks, followed by aspirin 81 mg monotherapy. A three-month follow-up CT angiogram appointment was scheduled, and the patient was advised to avoid chiropractic neck manipulations, roller coasters, heavy lifting, extreme exertion, and contact sports. The patient was admitted for pain management, improving with treatment from acetaminophen and oxycodone, and was discharged the following day.

## Discussion

The patient in this case presents with a complaint of headache radiating to the neck with no other symptoms nor significant physical exam findings. Headaches are a common non-specific complaint, and their cause can range from being mild and benign, such as a tension headache, to severe and life-threatening, such as SAH. In the absence of red-flag symptoms, headaches caused by VADs may go unnoticed during the initial visit, as seen in this case, unless the physician has a high index of suspicion and can reveal an inciting event in the patient's history. Typically, this type of headache is often occipital and ipsilateral to the causative injury, with the quality of pain varying from sharp to diffuse/constrictive pain [12]. There have been cases where it mimics other types of headaches. For instance, a thunderclap pain would require a differential diagnosis with a SAH [13]. On the other hand, posterior localization with a pressing quality may be mistaken for a tension-type headache [14], and although rare, migraine characteristics of visual aura (such as scintillating scotomas) being attributed to emboli in the occipital lobe have been reported [15]. What may provide a diagnostic hint would be an observation of any exacerbation or alleviation of the pain with variations in neck movements, such as flexion or extension [16]. Neck pain and headache are common clinical presentations of VAD, and accompanying neurological deficits may develop after a delay ranging from hours to days or even weeks [17]. Therefore, VAD should be considered in the differential diagnosis of headache and neck pain as early detection allows time for adequate treatment and avoiding major complications, such as stroke. 

Upon the patient's return to the ED due to worsening headache, further investigation revealed a history of two neck trauma-inducing events. These events included a chiropractic neck manipulation followed by an entire day of riding multiple roller coasters a couple of weeks later. Of note, it is estimated that one in 20,000 spinal manipulations has resulted in VADs [18]. It can be challenging to recognize VAD immediately due to its subtle presentation, often masked by pain, which may conceal disabling neurological deficits. With this in consideration, it may prove helpful for chiropractors to evaluate high-risk patients before treatment, especially those with connective tissue diseases, for clinical signs of VAD to prevent the risk of acute ischemic strokes [18,19]. Other proposed factors contributing to the risk of VAD include age, gender, history of migraines, smoking, hypertension, diabetes, oral contraceptive use, or cervical spondylosis. Interestingly, conflicting findings exist regarding patients with a prior increased stroke risk due to hypertension or diabetes mellitus, as they may not necessarily have an increased risk of stroke after spinal manipulation [20]. While it is commonly assumed that complications of neck manipulation can be reduced through early screening of high-risk patients and assessing vertebral artery patency, previous attempts to assess vertebral artery patency were inconclusive in identifying patients at an increased risk of VAD. Notably, after neck trauma or manipulation, the risk of stroke was found to be dependent on the manipulation technique and rotational forces applied to the neck [19]. While it was unclear per the initial review whether the risk of VAD was dependent on specific chiropractic techniques or the frequency of chiropractic treatments, there may be a potential correlation with a greater risk associated with increased applied rotational forces.

Another overlooked and unexpected risk factor in trauma-induced VAD includes roller coasters, with only a few case reports documenting it as an associated complication. With roller coasters, common neurological complications can include subdural hematomas, SAHs, cervicocephalic arterial dissection, carotid artery thrombosis with stroke, and posttraumatic migraine [10]. Given the increased force of gravity (G force) and intense extension/flexion of the neck experienced during roller coaster rides, both the carotid and vertebral intima are more vulnerable to tearing or dissection [10,21,22]. As a result, the aftermath of VAD is often characterized by non-specific symptoms such as dizziness, vertigo, neck pain, or headache, with ischemic stroke being the most commonly observed complication [23]. While ischemic strokes are generally rare, cervical artery dissections (carotid and vertebral artery) disproportionately contribute to 10-25% of ischemic strokes in younger adults less than 50 years old [24,9]. When examining our patient's medical history preceding their worsening headaches, we identified two significant risk factors, chiropractic neck manipulation and exposure to roller coasters, that occurred within several weeks. It is plausible that the patient experienced a compounded effect of both risk factors, potentially increasing their overall risk for developing VAD. This highlights the importance of considering diverse and compounded sources of neck trauma when assessing a patient who presents with non-specific symptoms of headaches and neck pain.

In summary, the diagnostic imaging with CT angiography and MR angiography revealed bilateral VAD, with the right side more severely affected than the left. Fortunately, early discovery and initiation of anticoagulation treatment played an important role in preventing the occurrence of ischemic stroke in the patient. Generally, for individuals diagnosed with VAD, close monitoring for neurological deficits and management through DAPT is essential in preventing strokes, a common complication of VAD [2]. While aspirin monotherapy is a standard preventative therapy for reducing vascular risk factors, a study found that dual treatment with clopidogrel and aspirin demonstrated significantly fewer ischemic events (such as ischemic stroke, myocardial infarctions, or death) and greater efficacy compared to aspirin monotherapy alone. However, it is noteworthy that DAPT was also associated with an increased risk of major hemorrhages compared to aspirin alone, warranting careful consideration when used [10]. Patients who survive the initial extracranial dissection (vertebral artery) have a favorable prognosis with around 80-90% recovering completely. Nevertheless, there remains an approximate 10% risk of recurrence, which could lead to stroke or possibly death. Conversely, patients who end up developing an intracranial dissection with neurological deficits or altered levels of consciousness tend to have a poorer prognosis. This is often linked to resulting infarctions in the brainstem, SAHs, or even death. It is important to also consider that the prognosis may vary for younger patients who may not have as many age-related comorbidities [2].

## Conclusions

This case emphasizes the significance of recognizing VAD as a potential cause of unexplained neck pain and headache, particularly in younger patients with prior exposure to trauma-inducing events. Due to the subtle presentation of VAD, often masked by pain, healthcare providers need to have a high index of suspicion for the early detection of VAD through MR angiography and CT angiography to prevent further complications like stroke. Due to the presentation of non-specific symptoms, as seen with our patient, it can be challenging to immediately diagnose VAD. Thus, a comprehensive history is crucial to elicit information regarding risk factors such as chiropractic manipulation and, unexpectedly, roller coaster rides in association with VAD. The compounded effect of both of these risk factors seen with our patient highlights the importance of considering various sources of neck trauma when assessing patients who present with non-specific symptoms. Additionally, with early detection, prompt initiation of DAPT can allow for proper management following treatment and improve overall patient outcomes and prognosis.
